# Isolation, purification and characterization of an ascorbate peroxidase from celery and overexpression of the *AgAPX1* gene enhanced ascorbate content and drought tolerance in *Arabidopsis*

**DOI:** 10.1186/s12870-019-2095-1

**Published:** 2019-11-11

**Authors:** Jie-Xia Liu, Kai Feng, Ao-Qi Duan, Hui Li, Qing-Qing Yang, Zhi-Sheng Xu, Ai-Sheng Xiong

**Affiliations:** State Key Laboratory of Crop Genetics and Germplasm Enhancement, Ministry of Agriculture and Rural Affairs Key Laboratory of Biology and Germplasm Enhancement of Horticultural Crops in East China, College of Horticulture, Nanjing Agricultural University, 1 Weigang, Nanjing, 210095 China

**Keywords:** Ascorbate peroxidase, Purification, Overexpression, *Arabidopsis*, Drought stress, Celery

## Abstract

**Background:**

Celery is a widely cultivated vegetable abundant in ascorbate (AsA), a natural plant antioxidant capable of scavenging free radicals generated by abiotic stress in plants. Ascorbate peroxidase (APX) is a plant antioxidant enzyme that is important in the synthesis of AsA and scavenging of excess hydrogen peroxide. However, the characteristics and functions of APX in celery remain unclear to date.

**Results:**

In this study, a gene encoding APX was cloned from celery and named *AgAPX1*. The transcription level of the *AgAPX1* gene was significantly upregulated under drought stress. AgAPX1 was expressed in *Escherichia coli* BL21 (DE3) and purified. The predicted molecular mass of rAgAPX1 was 33.16 kDa, which was verified by SDS-PAGE assay. The optimum pH and temperature for rAgAPX1 were 7.0 and 55 °C, respectively. Transgenic *Arabidopsis* hosting the *AgAPX1* gene showed elevated AsA content, antioxidant capacity and drought resistance. Less decrease in net photosynthetic rate, chlorophyll content, and relative water content contributed to the high survival rate of transgenic *Arabidopsis* lines after drought.

**Conclusions:**

The characteristics of APX in celery were different from that in other species. The enhanced drought resistance of overexpressing *AgAPX1* in *Arabidopsis* may be achieved by increasing the accumulation of AsA, enhancing the activities of various antioxidant enzymes, and promoting stomatal closure. Our work provides new evidence to understand APX and its response mechanisms to drought stress in celery.

## Background

Under abiotic stresses, plants generate numerous reactive oxygen species (ROS), including superoxide radical, hydrogen peroxide (H_2_O_2_), and lipid peroxides. Excessive accumulation of ROS damages plant cells via lipid peroxidation and protein oxidation [1, 2]. Many enzymatic and non-enzymatic antioxidants can reduce ROS levels to maintain cellular redox balance [1]. Plants contain various enzymatic antioxidants, such as superoxide dismutase (SOD), peroxidase (POD), catalase (CAT), and ascorbate peroxidase (APX), as well as non-enzymatic antioxidants, such as ascorbate (AsA), glutathione, flavonoids and carotenoids for ROS scavenging system [3, 4].

APX of the heme peroxidase superfamily [5, 6] is involved in the recycling pathway of AsA and environmental stress response in plants. AsA is a well-known plant antioxidant. Natural antioxidants have attracted increasing attention because of their beneficial effects on humans [7]. Plant resistance is also an enduring topic in the field of plant science [8, 9]. Several researchers focused on improving the ability of plants to tolerate abiotic stresses, including saline-alkali [10], drought [11], heat [12], and cold [13, 14]. APX is a key enzyme in the AsA–glutathione (AsA–GSH) pathway that removes excessive H_2_O_2_ in plant cells under normal and stress conditions [15]. APX catalyzes the reduction of H_2_O_2_ using AsA as an electron donor to water and monodehydroascorbate [16]. Then monodehydroascorbate spontaneously transforms into dehydroascorbate.

APX is widely studied because of its important roles in higher plants. It is also found in eukaryotic algae [17]. APX has been purified and studied in tea [18], rice [19], komatsuna [20], potato [21], and many other species. The physic-chemical properties of APXs vary in different species.

Drought is still the most vital constraint in crop production and food security, especially in areas where agricultural water resources are insufficient. Global warming and increasing droughts have aggravated the loss of the agricultural economy [22]. Many studies have reported that APX is involved in AsA accumulation and abiotic stresses such as salt stress [23–25]. However, related reports in resisting drought stress are poor.

Celery is a vegetable belonging to Apiaceae with rich nutritional value, and its growth is influenced by multiple abiotic stresses [26–28]. In our previous study, we determined changes in the expression of the *APX* gene during the developmental stages of celery [29]. However, the characteristics and transcriptional regulation mechanism of APX under drought stress in celery remain unknown. In the present study, the *AgAPX1* gene was cloned from celery, and its expression levels under simulated drought stress were detected. The active recombinant protein rAgAPX1 was obtained and then characterized. Furthermore, the subcellular localization of AgAPX1 was investigated. The AsA content, antioxidant capacity, and drought tolerance of *Arabidopsis* overexpressing *AgAPX1* were also detected and analyzed. The results of this study imply that AgAPX1 can enhance the drought tolerance and serve as a potential target for resistance breeding with gene engineering in celery.

## Results

### Nucleotide sequence and deduced amino acid sequence of AgAPX1

**Fig. 1:**
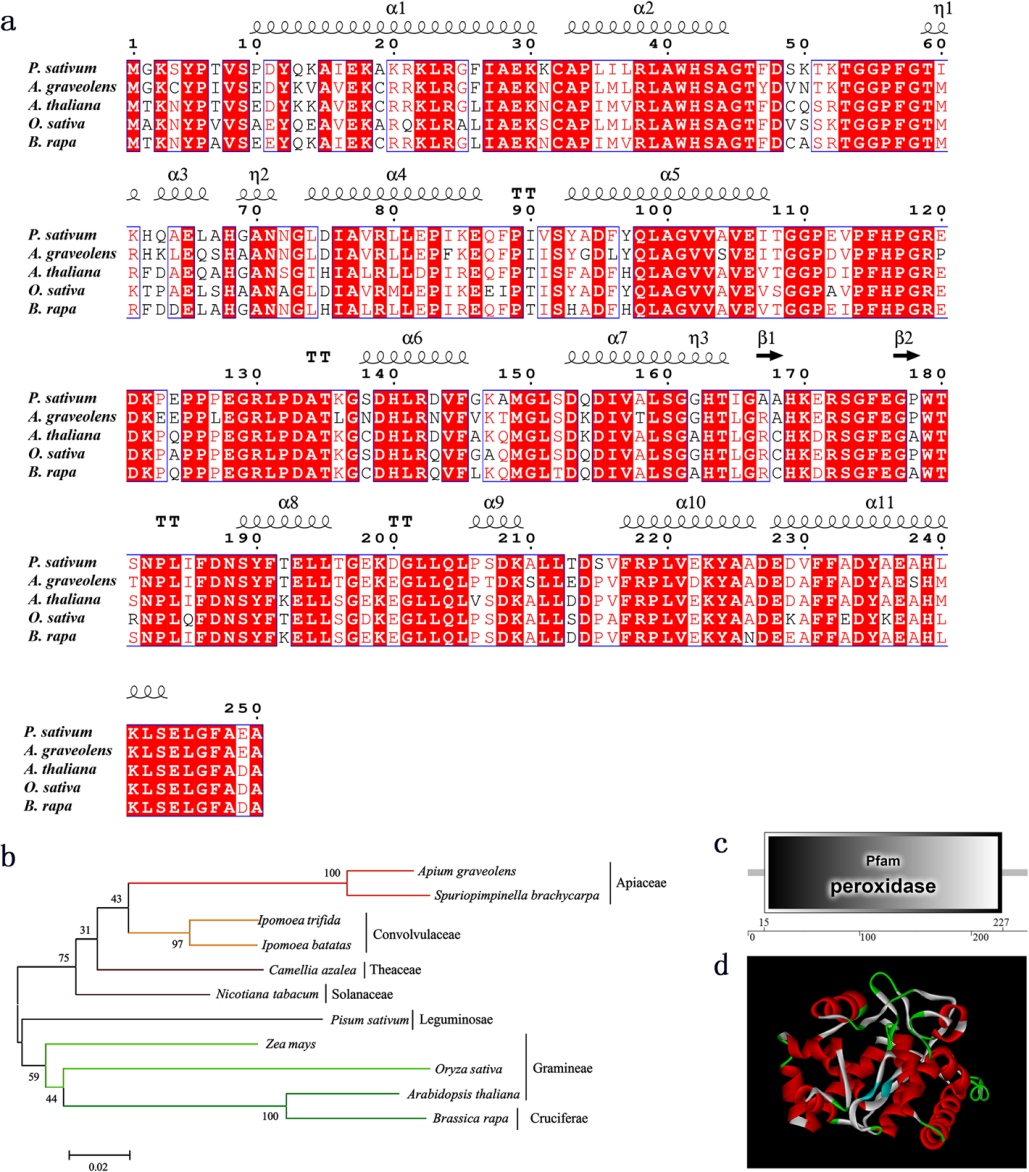
Sequence characteristic analysis AgAPX1 protein. **a** Alignment of deduced amino acid sequences of AgAPX1 with other ascorbate peroxidase from *Pisum sativum* (Accession No. P48534.2), *Arabidopsis thaliana* (Accession No. Q05431.2), *Brassica rapa* (Accession ACV92696.1), and *Oryza sativa* (Accession No. Q10N21.1) contained secondary structure elements (helices with squiggles, β-strands with arrows and turns with TT letters). **b** Phylogenetic relationships of deduced amino acid sequences of AgAPX1 and other APX proteins. *Arabidopsis thaliana* (Q05431.2), *Brassica rapa* (ACV92696.1), *Camellia azalea* (AKP06507.1), *Ipomoea batatas* (ALP06091.1), *Ipomoea trifida* (AGT80152.1), *Nicotiana tabacum* (AAA86689.1), *Oryza sativa* (Q10N21.1), *Pisum sativum* (P48534.2), *Spuriopimpinella brachycarpa* (AAF22246.1), and *Zea mays* (NP_001105500.2). **c** The predicted domain location of AgAPX1. **d** Three-dimensional structures of AgAPX1. The red and blue parts mark the alpha helix and beta turn, respectively

As shown in Additional file 1, *AgAPX1* cDNA consists of 753 bp nucleotides and encodes 250 amino acids. The molecular mass and theoretical *pI* of native AgAPX1 were 27.72 kDa and 5.41, respectively. The protein formula was C_1244_H_1926_N_332_O_371_S_8_ with a weak hydrophilicity. The deduced amino acid sequence of AgAPX1 was aligned with homologous sequences from other species, including *Pisum sativum*, *Arabidopsis thaliana*, *Brassica rapa*, and *Oryza sativa* using the ESPript 3.0 website [30]. The similarity of APX sequences between celery and *P. sativum* was the highest (80.8%) (Fig. 1a). Phylogenic tree showed that the APX proteins from *Apium graveolens* and *Spuriopimpinella brachycarpa* belonged to the same branch, and the APX proteins from other plants in same family were also clustered together (Fig. 1b). AgAPX1 and the APX proteins from other monocot plants were distant in evolution. In addition, the AgAPX1 protein has a specific peroxidase domain from sites 25 to 227 (Fig. 1c). Three-dimensional structure analysis using the pea protein (PDB ID:1apx) as template indicated that AgAPX1 contains thirteen TM α-helices and two β-sheets (Fig. 1d).

### Expression profiles of *AgAPX1* under PEG 6000 treatment in celery

**Fig. 2:**
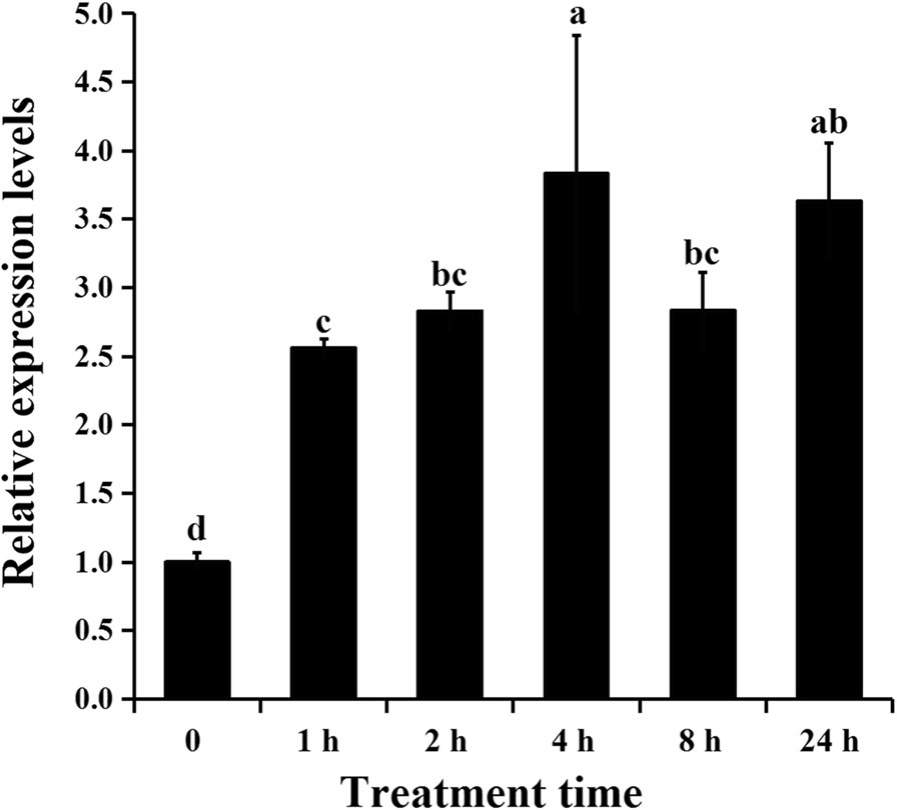
Expression profile of *AgAPX1* gene under drought stress in ‘Jinnanshiqin’. Different letters represent significant difference at 0.05 level

The expression profiles of *AgAPX1* were detected to verify its response under dehydration stress in celery. As shown in Fig. 2, the expression level of the *AgAPX1* gene at 1 h under PEG treatment was 2.56 folds higher than that of the control, and peaked at 4 h followed by a decrease. The results indicated that the *AgAPX1* gene responsed to PEG 6000 treatment and involved in dehydration stress in celery.

### Expression of AgAPX1 in *Escherichia coli* and purification of recombinant AgAPX1

The *AgAPX1* gene was cloned into the pET30 vector to construct a protein expression vector (pET-30a(+)-AgAPX1) and then expressed in *E. coli* BL21(DE3). A purified recombinant protein of AgAPX1 was prepared and named rAgAPX1. Its molecular weight was 33.16 kDa as calculated by ExPASy. Coomassie-stained SDS-PAGE of the purified rAgAPX1 protein showed a single band at about 34 kDa, which corresponds to the calculation (Additional file 2).

### Characterization of rAgAPX1

**Fig. 3:**
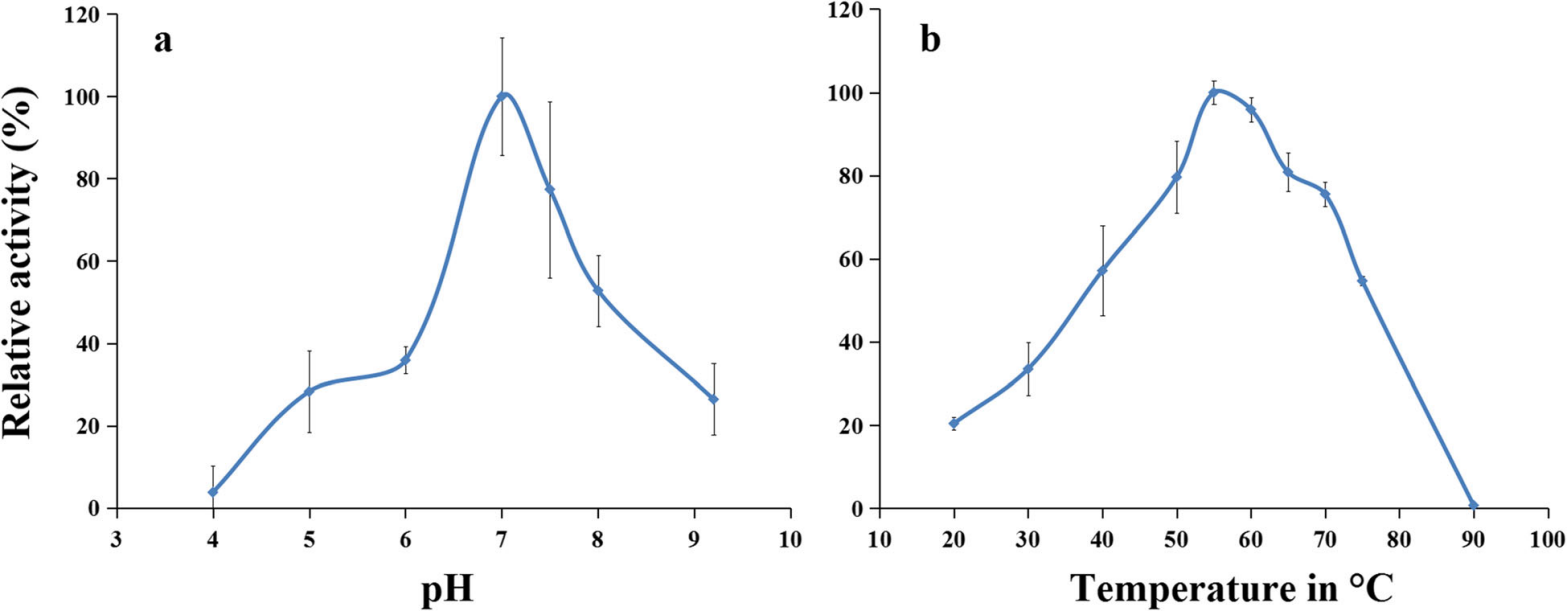
Effect of pH (**a**) and temperature (**b**) on purified AgAPX1 activity

The optimum pH for the rAgAPX1-catalyzed oxidation of AsA was pH 7.0 (Fig. 3a). The relative enzyme activity of rAgAPX1 was low in the acid environment, maintained a rising activity until pH 7.0, and then decreased. The result indicates that the suitable reaction environment of the enzyme is neutral. The optimum temperature for rAgAPX1 was found to be 55 °C (Fig. 3b). The enzyme activity of rAgAPX1 increased with reaction temperature from 20 °C to 55 °C, peaked at 55 °C followed by a decrease, and then deactivated at 90 °C.

### Subcellular localization of the AgAPX1 protein

**Fig. 4:**
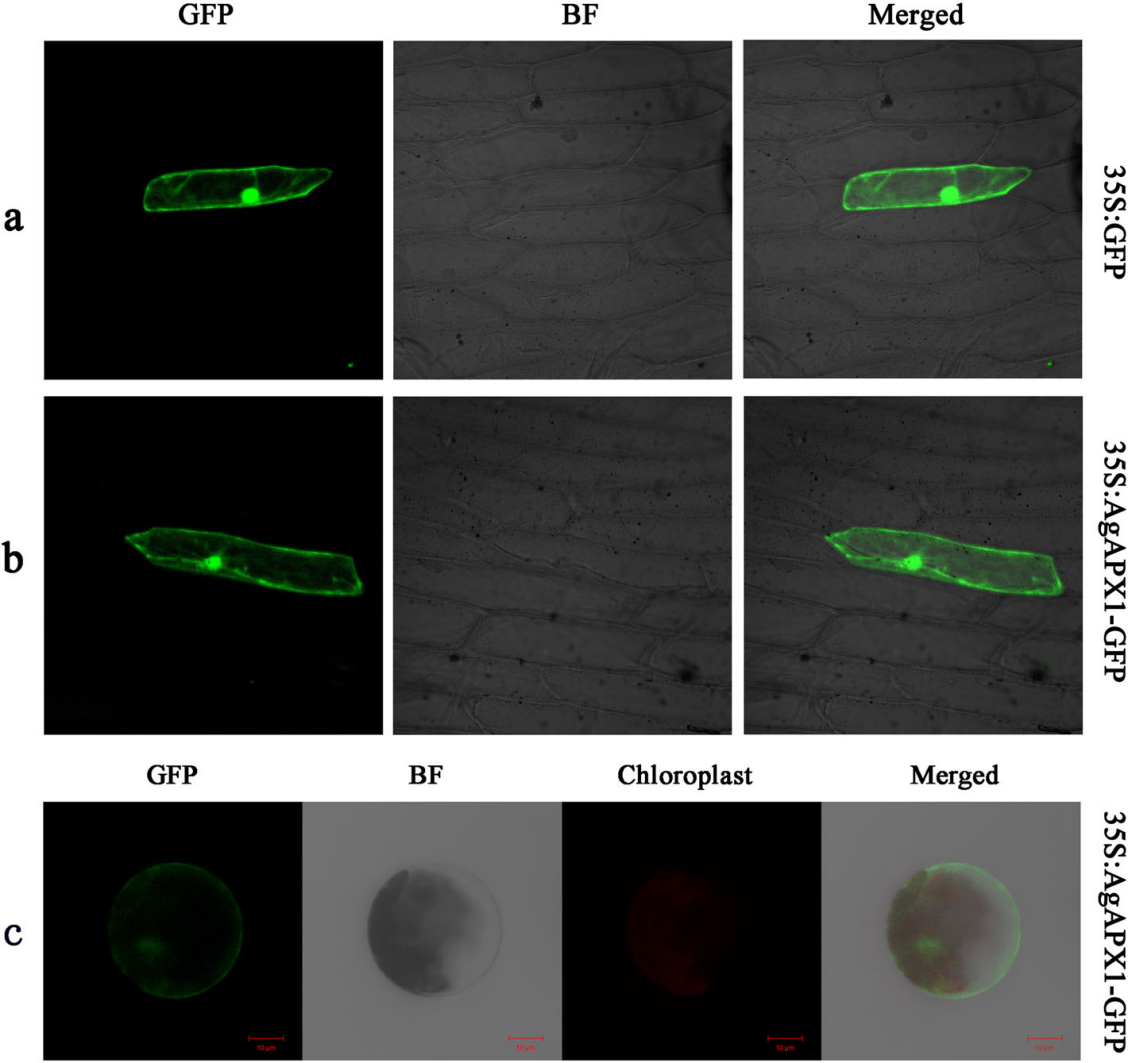
Subcellular localization analysis of AgAPX1. **a** Control vector (pA7-GFP) expressed in onion epidermal cells. **b** Recombinant vector (AgAPX1-GFP) expressed in onion epidermal cells. **c** AgAPX1-GFP fusion proteins transiently expressed in *Arabidopsis* mesophyll protoplasts. GFP: green fluorescent protein; BF: bright field; chloroplast: chlorophyll auto fluorescence; merged: combined fluorescence from GFP, BF, and chloroplast

The empty vector (pA7-GFP) and recombinant vector (AgAPX1-GFP) were expressed in onion epidermal cells to investigate the subcellular localization of AgAPX1. The onion epidermal cell with empty vector displayed strong fluorescence throughout the entire cell (Fig. 4a). Meanwhile, the onion epidermal cells transformed by the recombinant vector showed a similar distribution of green fluorescence to the empty vector (Fig. 4b). The 35S:AgAPX1-GFP fusion-construct was also expressed in *Arabidopsis* mesophyll protoplasts. The green fluorescence signals were mainly displayed in the nucleus and the plasma membrane, and did not overlap with chloroplast fluorescence (Fig. 4c). These results suggest that AgAPX1 is located in the nucleus and membrane and not in the chloroplast.

### Heterologous expression of *AgAPX1* in *Arabidopsis* increased the AsA content and total antioxidant capacity

**Fig. 5:**
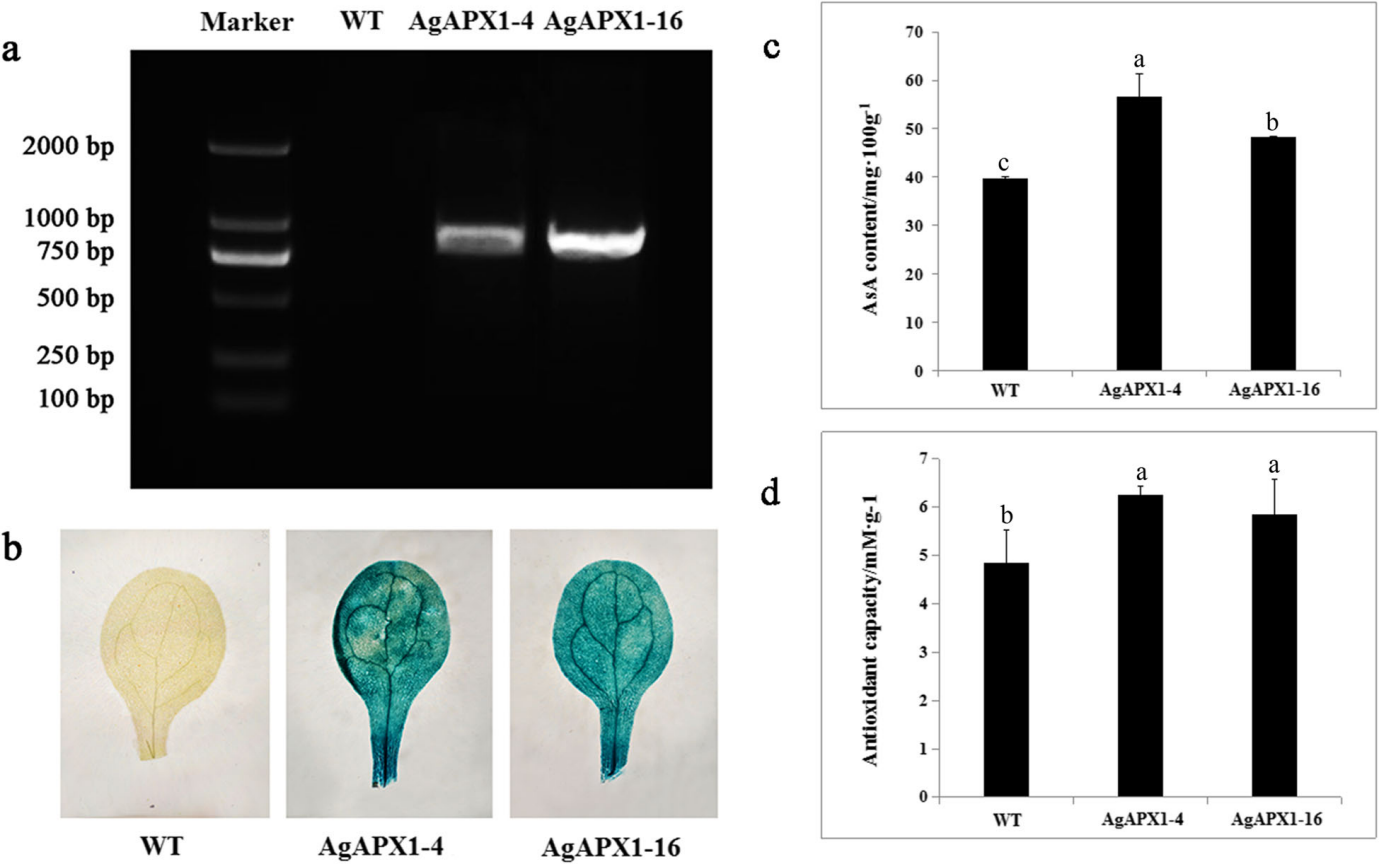
PCR amplification, GUS stain identification, and functional analysis of *Arabidopsis* transformed with a *AgAPX1* gene. **a** PCR amplification of *AgAPX1* from the cDNA of transgenic *Arabidopsis* plants. **b** Histochemical GUS assays of control line and transgenic lines. **c** Ascorbate levels of 4-weeks-old transgenic *Arabidopsis* and WT plants. **d** The difference of antioxidant capacity between 4-weeks-old transgenic *Arabidopsis* and WT plants. WT, non-transgenic *Arabidopsis* (negative control); AgAPX1–4 and AgAPX1–16, transgenic *Arabidopsis* plants. Error bars represent standard deviation among three independent replicates. Data are expressed as the means ± standard deviation (SD) of three replicates. Different letters represent significant difference at 0.05 level

Transgenic *Arabidopsis* plants overexpressing the *AgAPX1* gene were generated to further investigate the molecular functions of *AgAPX1*. The positive transgenic lines hosting the *AgAPX1* gene were confirmed by gene-specific amplification (Fig. 5a). Two transgenic lines showed blue by β-glucuronidase (GUS) staining (Fig. 5b). AsA levels were assessed using 4-week-old *Arabidopsis* leaves by HPLC. The AsA contents in the two transgenic lines were markedly higher than that in the wild-type (WT) *Arabidopsis* plants (Fig. 5c and Additional file 3). The AgAPX1–4 line had the highest level of AsA, which was approximately 1.4 times higher than that in the WT plants. The total antioxidant capacities of the AgAPX1–4 and AgAPX1–16 lines increased by 29.10 and 21.16% as evaluated by FRAP assays, respectively (Fig. 5d).

### Overexpression of *AgAPX1* in *Arabidopsis* positively regulates drought tolerance by regulating the stomata aperture

**Fig. 6:**
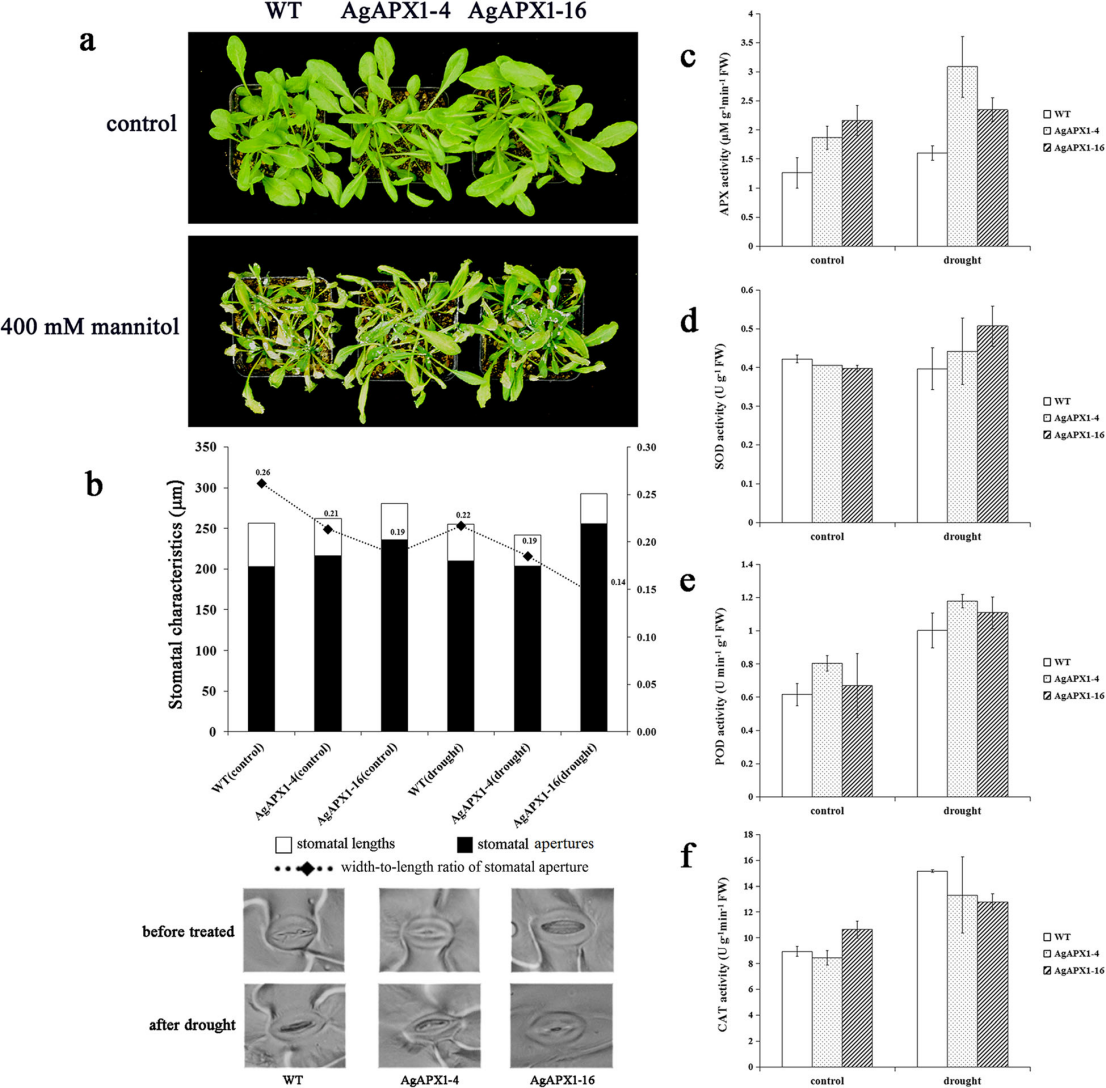
Phenotype, stomata state and activity of antioxidant enzymes in wild-type (WT), transgenic lines (AgAPX1–4 and AgAPX1–16) before and after drought treatment. **a** Comparison of growth phenotype among transgenic and non-transgenic *Arabidopsis* lines. For drought stress treatments, these plants were treated with 400 mM mannitol and photographed after 7 days. **b** Stomatal aperture measurements of different *Arabidopsis* lines in response to 24 h of drought. **c** APX activity assay (after 24 h of treatment). **d** SOD activity assay (after 24 h of treatment). **e** POD activity assay (after 24 h of treatment). **f** CAT activity assay (after 24 h of treatment)

Transgenic and WT *Arabidopsis* plants were treated with 400 mM mannitol to study the tolerance to drought stress. The phenotype of *Arabidopsis* plants was observed after 7 days of mannitol treatment (Fig. 6a). The transgenic lines showed a better status under drought condition. The stomatal apertures of WT and transgenic lines both decreased in response to stress. The two transgenic *Arabidopsis* lines showed smaller stomatal apertures after 24 h of drought treatment. The width-to-length ratio of stomatal aperture in the two transgenic *Arabidopsis* plants decreased to 0.85 and 0.66 times as much as that in the WT plants (Fig. 6b).

### Analysis of antioxidant enzyme activities in transgenic lines before and after treatment

The activities of four key enzymes, APX, SOD, POD, and CAT, were measured in WT and two transgenic *Arabidopsis* lines under normal condition and drought treatment. APX, SOD, and POD are major enzymes in the ROS scavenging system. CAT is also an important antioxidant enzyme in plants [31]. In plant life cycle, H_2_O_2_ accumulates under various abiotic stresses [1]. Excessive H_2_O_2_ oxidizes biological macro-molecules (nucleic acids and proteins) directly or indirectly, and damages cell membranes, thus accelerating cell senescence and disintegration. CAT can scavenge H_2_O_2_ and maintain the balance of ROS metabolism in plants. The activity of CAT in plant tissues is also closely related to plant stress resistance [31, 32].

The transgenic *Arabidopsis* lines had significantly higher APX activities than the WT plants (Fig. 6c). The APX activities of all samples increased after drought treatment, and the increase was more remarkable in the AgAPX1–4 transgenic line. In addition, drought stress enhanced the SOD activities in the two transgenic lines compared with the WT plants (Fig. 6d). The POD activities in all of the tested samples increased after drought stress (Fig. 6e). However, the CAT activities in the WT plants showed markedly increased than those in the two transgenic lines after drought treatment (Fig. 6f).

### Physiological changes in *Arabidopsis* leaves exposed to drought stress

**Fig. 7:**
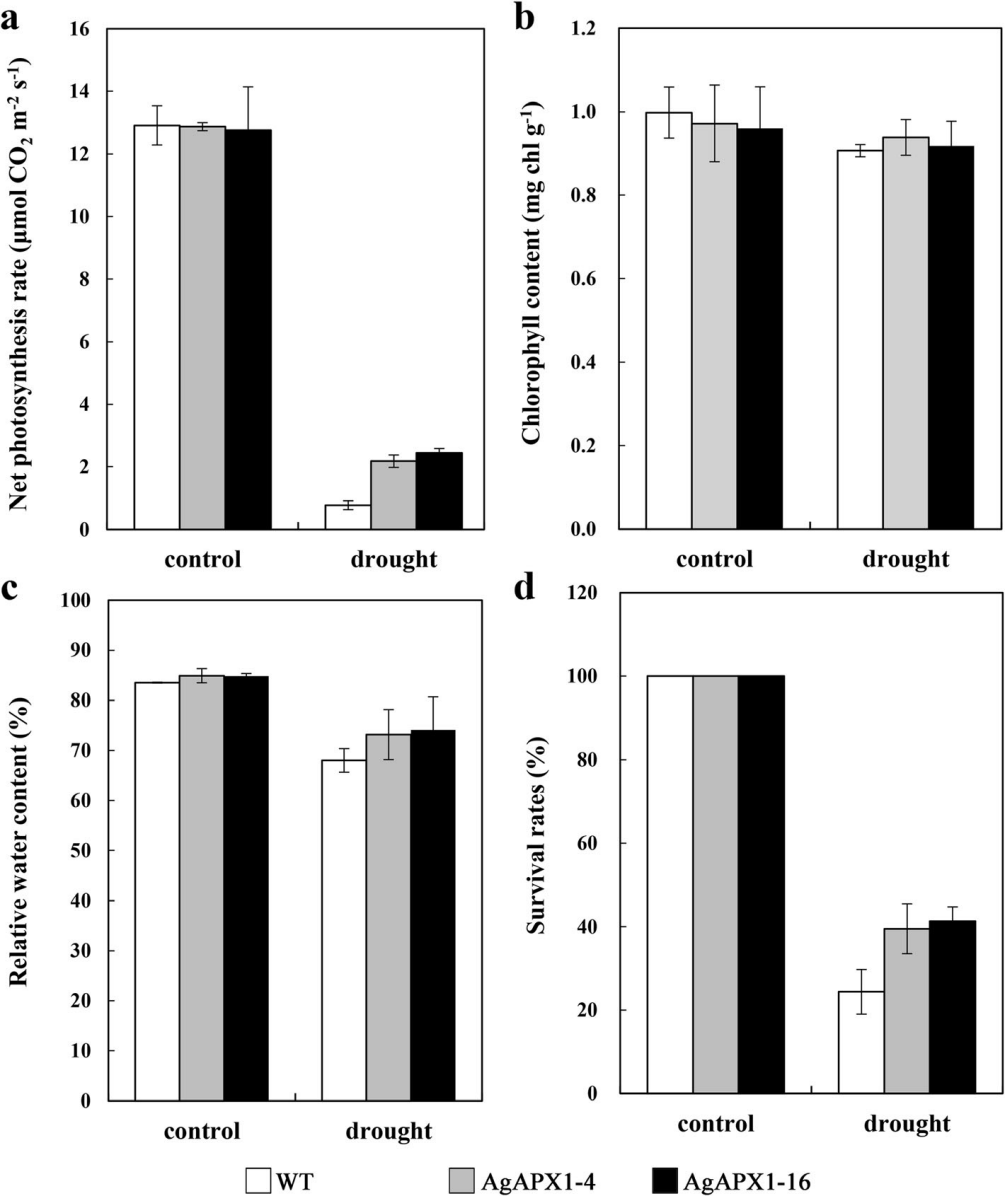
Physiological changes in the leaves of wild-type (WT) and transgenic *Arabidopsis* lines (AgAPX1–4 and AgAPX1–16) subjected to drought. **a** Net photosynthetic rate. **b** Total chlorophyll content. **c** Relative water content. **d** Plant survival rate

The net photosynthetic rate (Pn) was measured to evaluate the photosynthetic activity in WT and transgenic *Arabidopsis* under drought stress. As shown in Fig. 7a, Pn considerably decreased in the WT over transgenic lines under drought stress. The results suggested that the photosynthetic system of WT incurred more severe damage than that of the transgenic lines. Total chlorophyll and relative water content (RWC) also considerably decreased in the WT plants compared with the transgenic lines (Fig. 7b and c). The survival rate was used to further demonstrate the drought tolerance of transgenic lines, and results showed that the two transgenic lines retained higher survival rate after treatment (Fig. 7d).

## Discussion

The APX protein contains hemoglobin that possesses a high specificity to ascorbic acid. APX also has a higher affinity with H_2_O_2_ and utilizes AsA as a specific electron donor, which plays a crucial role in modulating or eliminating H_2_O_2_ from cells and maintaining cellular redox equilibrium [33–35]. Celery, which is rich in AsA, is widely cultivated worldwide [29]. In the present work, the *AgAPX1* gene, was cloned from celery cv. ‘Jinnanshiqin’. The prediction of AgAPX1 specific structure will help us understand its particular function. AgAPX1 is highly conserved and characterized with a peroxidase domain. The deduced amino acid sequence of AgAPX1 was the highest similar to that of APX from *P. sativum*. AgAPX1 is very conservative and shows a high homology to other APXs. Phylogenic analysis indicated that the APX protein of the same family is closer in evolution and the sequence divergence of APX might occur after the monocot–dicot split as other structural genes [36].

Several reports reported the purification of APX from plant tissues or *E. coli* cells to investigate its functions [19, 20, 37]. Previous studies indicated that the purified APX has a molecular mass ranging from 28 kDa to 31 kDa [20, 21, 38]. The molecular mass of rAgAPX1 from celery was higher, which was approximately 34 kDa as measured by SDS-PAGE. The optimal temperature and pH of the rAgAPX1 enzyme were 55 °C and 7.0, respectively. By contrast, the optimal cytosolic APX activity was at 38 °C and pH 6.5 in komatsuna [20]. In liverwort, the APX enzyme had an optimal temperature of 40 °C and an optimal pH of 6.0 [38]. The differences in physicochemical properties among different species may be caused by the differences in APX gene sequence. Another reason may be affected by the insertion of His-tag, S-tag, enterokinase, and thrombin sequences in the N-terminus of the rAgAPX1 protein [39, 40].

The analysis of gene expression is an important strategy to understand the molecular mechanisms of stress responses in higher plants. Previous studies have reported that plants generally exhibited increased APX activity under stress conditions, which is usually correlated with increased stress tolerance [41, 42]. Simultaneously, the expression of *APX* genes may be induced to protect against oxidative stress [42]. The activity of APX enzymes is enhanced when subjected to cold and salt stresses [16, 43]. In addition, the expression of the *APX* gene is upregulated under drought stress in *Eleusine coracana* [44]. In the present study, the transcription level of the *AgAPX1* gene was markedly upregulated when exposed to PEG-induced dehydration stress in ‘Jinnanshiqin’ plants, which is consistent with the results of previous researches [45, 46]. The result suggests that the *AgAPX1* gene involved in the response of celery to drought stress. The response of the *AgAPX1* gene to adversity may be attributed to the conservation of APX sequences among different species. In addition, most of the differentially expressed genes related to antioxidant enzymes are localized in the mitochondria, chloroplast, and peroxisome [47, 48]. APXs have several forms of isoenzymes according to their distribution in plant cells, including stromal (sAPX), thylakoid membrane (tAPX) in the chloroplast, microbody APX (mAPX), and cytosolic APX (cAPX) [17]. Our study indicated that AgAPX1 is mainly localized in the nucleus and membrane. The probable functions of APX in the nucleus are to orchestrate ROS signaling and regulate related genes expression, thereby regulating plant physiological changes and stress response [49, 50].

Drought stress is a global problem that limits horticultural productivity. Some defense mechanisms in plants are aroused under stress to protect themselves from the damage of oxidative stress [51]. Previous studies also reported that the overexpression of *APX* in plants enhances the tolerance to both cold and heat stresses [50]. Overexpression of the *SsAPX* gene isolated from *Suaeda salsa* in *Arabidopsis* protects plants against salt-induced oxidative stress [52]. AsA is a natural antioxidant and acts directly to neutralize superoxide radicals in plant response to abiotic stress [24]. The overexpression of genes involved in AsA metabolism, such as *GMP* [36], *GGP* [53], and *DHAR* [54], provides an elevated resistance to abiotic stresses by regulating the generation of AsA in plants. We found that overexpressing *AgAPX1* in *Arabidopsis* showed increased AsA content significantly. Under drought stress, the transgenic *Arabidopsis* (AgAPX1–4 and AgAPX1–16) lines displayed better growth status than the WT plants. Multiple enzymatic antioxidants play key roles in scavenging the toxic ROS and protecting the plants from oxidative damage [55]. The enzyme activity of APX in transgenic *Arabidopsis* also increased. AgAPX-16 line showed higher APX activities than AgAPX-4 line. The possible reason is that AsA biosynthesis is regulated complexly by many factors aside from APX activity [29]. Drought stress induced a prominent increase in the activities of some antioxidant enzymes, including APX, SOD, and POD in the transgenic *Arabidopsis* plants. These results suggest that transgenic *Arabidopsis* plants hosting *AgAPX1* had an increased tolerance to drought stress by enhancing AsA accumulation and the activities of antioxidant enzymes. AgAPX1–16 line showed a lower increment of APX activity than the AgAPX1–4 line in response to drought. The difference in APX activity of transgenic lines may be associated with insertion site, post-transcription, and post-translation of the target gene. The WT plants showed a greater elevation of CAT activity than the transgenic plants. CAT, a direct H_2_O_2_ scavenging enzyme, was used to eliminate excessive H_2_O_2_ generated from drought stress in the anti-oxidative defense system of plants [56].

The increased stress tolerance is also probably associated with the closure of stomata in plants. The key role of stomatal closure in plant innate immunity has been described [57]. In higher plant, abiotic stress could induce stomatal closure. CO_2_ levels are decreased in the leaves, which promotes oxygenation of RuBP to produce additional H_2_O_2_ [58, 59]. ROS species such as H_2_O_2_ are mainly targeted by APXs [33]. Therefore, the decreased stomatal apertures and elevated antioxidant enzyme activities of the transgenic *Arabidopsis* plants under drought stress are justifiable. Meanwhile, the drought-induced reduction in net photosynthetic rate, relative water content, and chlorophyll content was much lower in the transgenic lines than in the WT plants. These results agree with previous reports [60, 61]. Less chlorophyll degradation and decreased lipid peroxidation have been detected in transgenic plants overexpressing *APX* gene under several adversities in previous studies [62, 63]. We concluded that the change in *AgAPX1* transcriptional level is an important regulatory mechanism in response to abiotic stress in celery and transgenic *Arabidopsis*.

In addition, gene silencing and knockout are important technical means to verify the function of genes in plants. Gene silencing is usually achieved using RNA interference (RNAi) [64]. And the CRISPR/Cas9 system has been used routinely in the knockout of genes [65]. However, the two technologies are both performed based on the transgenic technology and an efficient transformation system has not yet been established in celery. We will focus on eliminating this limitation to generate stable loss-of-function plants for further functional verification in our future work.

## Conclusion

A key enzyme of AsA–GSH cycle in celery, AgAPX1, was identified and characterized in this study. The current results provided important information on the potential characteristics of the APX enzyme. The response of *AgAPX1* to drought in celery and the heterologous expression of the *AgAPX1* gene in *Arabidopsis* further elucidated the important roles of AgAPX1. *AgAPX1* transgenic lines may establish a reference for selecting crop plants with high drought tolerance and increase the confidence and basis of its homologous expression by transgenic technology to enhance stress tolerance in celery. These findings are important for farming in areas under drought stress.

## Methods

### Plant materials and stress treatment

*Arabidopsis* Columbia ecotype (the control *Arabidopsis*) and celery cv. ‘Jinnanshiqin’ were deposited at the State Key Laboratory of Crop Genetics and Germplasm Enhancement, Nanjing Agricultural University (32°04′N, 118°85′E). The plants were grown in pots containing a mixture of organic soil and vermiculite (3:1, v/v) in an artificial climate chamber (a 16-h photoperiod, an illumination of 240 μmol m^− 2^ s^− 1^, day/night temperatures of 25/16 °C and a relative humidity of 70%). For dehydration stress, two-month-old celery plants were treated with 20% PEG 6000. The leaf blades of ‘Jinnanshiqin’ were harvested after 1, 2, 4, 8, 12, and 24 h and immediately frozen in liquid nitrogen and stored at -80 °C until analysis. The *Arabidopsis* seeds were sown on MS solid medium for 7 days, transferred to a commercial potting soil mixture, and then placed in a controlled growth chamber at 22 °C with a relative humidity of 70% under the condition (12 h: 12 h, light: dark). Four-week-old *A. thaliana* seedlings were irrigated with 400 mM mannitol solution. The samples were collected at 24 h after treatment for further analysis.

### Gene searching, cloning, and bioinformatics analysis

The amino acid sequences of the DcAPX (Accession No. AKH49594.1), an APX from carrot, was used to blast against our celery transcriptome database to obtain the gene encoding APX [66, 67]. The open reading frame (ORF) in comp120_c0_seq1 was the most similar to the sequence of DcAPX. This ORF was designated as *AgAPX1* in this study. The gene was cloned from the cDNA of ‘Jinnanshiqin’ celery by PCR amplification. The cloning primers of *AgAPX1* were 5′-ATGGGAAAGTGCTATCCAATTGT-3′ and 5′-TTAGGCCTCAGCAAACCCAAGT-3′. Then, the basic physical and chemical properties of the predicted protein were analyzed using ExPASy (http://expasy.org/tools/). The functional domains of the AgAPX1 protein were analyzed using the SMART program. The three-dimensional structure modeling was performed using CPHmodels 3.2 Server (http://www.cbs.dtu.dk/services/CPHmodels/) online analysis software. The amino acid sequences of APX proteins from different species were aligned in the Clustal X program, and a phylogenetic tree was constructed using MEGA 6.0 [68].

### Expression patterns of *AgAPX1* gene

The RT-qPCR system was performed on the CFX96 Real-Time PCR system (Bio-Rad) with SYBR Premix *Ex Taq*. RNA was extracted using the *RNAprep* pure plant kit (Tiangen-bio, Beijing, China) in accordance with the manufacturer’s instructions, followed by reverse transcription to cDNA. A 15 × diluted cDNA sample was used for RT-qPCR analysis. The *AgActin* gene was used as an internal standard for normalization [69]. The primer pairs of the *AgAPX1* gene for RT-qPCR were 5′-GCCGCTTGCCTGATGCTACTT-3′ and 5′-CCTTCAAACCCAGAACGCTCCTT-3′. The relative expression data were calculated using the 2^−∆∆Ct^ method [70]. Each sample was performed with three biological replicates.

### Expression of AgAPX1 in *E. coli*

The *AgAPX1* gene was cloned into the vector pET-30a (+) between the *Bam* HI and *Sac* I sites. The recombinant forward primer and reverse primer were 5′-GCCATGGCTGATATCGGATCCATGGGAAAGTGCTATCCAATTGT-3′ and 5′-GCAAGCTTGTCGACGGAGCTCTTAGGCCTCAGCAAACCCAAGT-3′, respectively. The amplified vector was then transformed into *E. coli* DH5α and confirmed by DNA sequencing. *E. coli* BL21(DE3) cells (TransGen, Beijing, China) were used for the expression of pET-30a (+)-AgAPX1. The transformed bacterial cells were grown in 50 mL of LB medium containing 50 mg·L^− 1^ kanamycin at 37 °C for about 4 h with 230 rpm shaking until the OD_600_ value reached 0.4–0.6. The recombinant protein was induced by adding isopropyl β-D-1-thiogalactopyranoside (IPTG) to a final concentration of 1.0 mM at 18 °C, and the culture was continued to shake (220 rpm) for over 12 h.

### Purification of recombinant protein AgAPX1

All steps of the recombinant protein purification were carried out at 4 °C. The cells were harvested by centrifugation; suspended in 4 mL of lysis buffer (pH = 7.5) containing 50 mM NaH_2_PO_4_, 300 mM NaCl, 10% glycerol, 10 mM β-mercaptoethanol and 10 mM imidazole; and disrupted by sonication for 20 min on ice. Cell homogenate after sonication was centrifuged at 12,000 rpm for 10 min at 4 °C, and the supernatant was filtered using 0.22-μm microfiltration membranes. The mixture was loaded on a column containing Ni-NTA-agarose resin (1.5 mL bed volume) (Qiagen, Hilden, Germany) equilibrated with equilibrium buffer (50 mM NaH_2_PO_4_, 300 mM NaCl, 10% glycerol and 10 mM imidazole, pH = 7.5). Washing with a buffer (pH = 7.5) containing 50 mM NaH_2_PO_4_, 300 mM NaCl, 10% glycerol, 10 mM β-mercaptoethanol, and 50 mM imidazole was performed more than six times to elute other proteins. Then, the His-tagged AgAPX1 was dissolved by elution buffer (50 mM NaH_2_PO_4_, 300 mM NaCl, 10% glycerol, 10 mM β-mercaptoethanol and 10 mM imidazole, pH = 7.5) and subsequently washed with equilibrium buffer to re-equilibrate the column. The purified enzyme was collected as previously described and then stored at -20 °C for further analysis. The purity of the active fractions was tested using 12% (w/v) SDS-PAGE as described by Laemmli [71] and then stained with Coomassie Brilliant Blue.

### AgAPX1 activity assay

APX activity was measured as previously described by Nakano & Asada (1987) [72] with some modifications. Briefly, the reaction mixture contained 50 mM PBS (pH = 7.0) with 0.1 mM EDTA-Na_2_, 0.5 mM AsA and 0.1 mM H_2_O_2_, and about 50 μg purified rAgAPX1 in a final volume of 300 μL. The reaction was initiated with the addition of H_2_O_2_. Enzyme activity was determined by measuring the decrease in absorbance at 290 nm (Ɛ_290_/2.8 mM^− 1^ cm^− 1^) due to AsA oxidation over a few minutes. Protein concentration was determined with Coomassie Brilliant Blue G-250 according to the method of Bradford using bovine serum albumin (BSA) as the standard.

### Effect of pH and temperature on AgAPX1 activity

The optimum pH for enzyme activity was determined in 50 mM citric acid–Na_2_HPO_4_ (pH = 4.0–5.0), 50 mM NaH_2_PO_4_–Na_2_HPO_4_ (pH = 6.0–8.0), and 50 mM glycine–NaOH (pH = 9.2). The effect of temperature (20 °C–90 °C) on enzyme activity was determined at pH 7.0 in 50 mM PBS for 5 min.

### Subcellular localization

The full-length sequence of the *AgAPX1* gene without the terminator was amplified and then inserted into the GFP-fusion expression vector pA7. The recombinant vector (AgAPX1-GFP) and empty vector (pA7-GFP) were transferred into onion cells with gold powder via a biolistic procedure by using a helium driven particle accelerator (PDS-1000, Bio-Rad). The onion epidermal cells were spread on the MS solid medium for over 16 h in a dark incubator at 25 °C. Moreover, the recombinant vector 35S:AgAPX1-GFP was transformed into *Arabidopsis* mesophyll protoplasts to identify the real location of AgAPX1 since some APXs are located in chloroplast and onion epidermal cells lack chloroplasts. The isolation of *Arabidopsis* mesophyll protoplasts and transient gene expression were conducted according to PEG-mediated transformation method described by Yoo et al. [73] with some modifications. The transformed protoplasts also were incubated in a dark incubator at 25 °C for over 16 h. The fluorescence signal of GFP fusion proteins and red chloroplast auto fluorescence were observed using a LSM780 confocal microscopy imaging system (Zeiss Co., Oberkochen, Germany).

### Overexpression vector construct and *Arabidopsis* transformation

The full-length ORF of *AgAPX1* was amplified using the specific primers (forward: 5′-TTTACAATTACCATGGGATCCATGGGAAAGTGCTATCCAATTGT-3′ and reverse:5′-ACCGATGATACGAACGAGCTCTTAGGCCTCAGCAAACCCAAGT-3′) and subcloned into the vector pCAMBIA-1301. The construct was verified by PCR and sequencing. The recombinant vector (CaMV 35S:AgAPX1) containing a 35S: GUS reporter gene sequence was introduced in the *Agrobacterium tumefaciens* strain GV3101 via electroporation. Transformation of WT plants was performed using the floral-dip method as previously described [74]. The transgenic lines of *Arabidopsis* were screened on a MS medium containing hygromycin (40 mg/L) and then planted. The seeds were then harvested. To further verify the presence of *AgAPX1* in the transgenic *Arabidopsis*, GUS staining and PCR amplification were conducted. The independent transgenic *Arabidopsis* lines obtained by screening were used in the next experiment.

### Determination of AsA content

AsA content was determined in accordance with the method described by Duan with slight modifications [75]. Briefly, about 0.3 g fresh leaves from transgenic and WT *Arabidopsis* plants were ground with 2 mL 1% (w/v) pre-cooling oxalic acid. The homogenate was centrifuged at 12,000 rpm for 10 min at 4 °C and the supernatant was filtered through a 0.45 μm membrane syringe filter. Sample analysis was performed using the Agilent 1200 HPLC system for assays of AsA levels at a wavelength of 245 nm. The mobile phase consisted of 0.1% (v/v) acetic acid at a flow rate of 1 ml/min. AsA content was expressed as mg/100 g (fresh weight, Fw).

### Antioxidant capacity analysis

The antioxidant capability of the samples was estimated by the FRAP (ferric reducing antioxidant potential) method. It was performed using the T-AOC Assay Kit (S0116, Beyotime, Shanghai, China) in accordance with the manufacturer’s instructions. Approximately 0.2 g fresh leaves of *Arabidopsis* plants were homogenated with 1 mL PBS (pH = 7.0), and the absorbance was monitored using a spectrophotometer at 593 nm. The result of antioxidant activity was recorded as mM Fe^2+^ per g fresh weight (mM Fe^2+^/g Fw).

### Analysis of stomatal characteristics

Images of the stomata were obtained using the abaxial epidermis of leaves detached from 4-week-old *Arabidopsis* plants before and after 24 h of drought stress. The observation and statistics of stomatal lengths and apertures were accomplished randomly using electron microscopy and CellSens image software.

### Crude enzyme extract and enzymatic activity measurement

The crude enzyme was extracted from 4-week-old leaves of both transgenic and non-transgenic *Arabidopsis* plants after 400 mM mannitol for 24 h. For SOD and POD activity determination, about 0.2 g fresh samples were extracted with 50 mM PBS (pH 7.8) and detected using Wang’s method [76]. For the determination of APX and CAT activities, fresh *Arabidopsis* leaves were homogenated with 50 mM PBS (pH 7.0, containing 0.1 mM EDTA). The test of crude APX activity was performed the same as that described above. CAT activity assay was performed following the modified method of *Sahu* et al. [77]. Each experiment was performed with three independent replicates.

### Measurements of photosynthetic rate, total chlorophyll content, RWC, and plant survival rate

Pn was measured using the Li-6400 portable photosynthesis system. Total chlorophyll content was determined following the method of Lichtenthaler and Wellburn [78]. RWC was measured as previously described [79]. It was calculated according to the formula as follow: RWC = [Fw-Dw]/[Tw-Dw]X100, where Fw is fresh weight of leaf, and Tw is rehydrated weight of leaf after incubating in water for 8 h, and then drying them in an oven at 80 °C until constant weight is recorded as Dw. In addition, the *Arabidopsis* plants were treated by withholding watering for 10 d, and then the survival rate was recorded after rewatering for 3 d after recovery. All the above experiments were carried out using 4-week-old leaves.

### Statistical analysis

All values reported in this study were the means of three independent replicate measurements, unless otherwise stated. The data were expressed as mean ± standard deviation (SD) of three replicates. Statistical significance of the differences was analyzed by SPSS 20.0 software (SPSS Inc., Chicago, IL, USA) using Duncan’s multiple-range test with a significance level of 0.05 (*P < 0.05*).

## Supplementary information

**Additional file 1 **Nucleotide acid and deduced amino acid sequence of *AgAPX1* from celery.

**Additional file 2 **SDS-PAGE analysis of the purified AgAPX1 from expression in *E. coli*. Lane1, standard markers; Lane2, total soluble protein from BL21(DE3) cells containing the AgAPX1 plasmid without induction; Lane3, total soluble protein from BL21(DE3) cells containing the AgAPX1 plasmid with IPTG; Lane 4, purified AgAPX1. The arrow indicates the AgAPX1.

**Additional file 3 **Ascorbate content in transgenic *Arabidopsis* and wild-type (WT) leaves detected by HPLC. **a** WT plants; **b** AgAPX1–4 transgenic line**; c** AgAPX1–16 transgenic line.

## Data Availability

The data sets supporting the conclusions of this article are included within the article and its additional files. *Arabidopsis* Columbia ecotype (the control *Arabidopsis*) and celery ‘Jinnanshiqin’ were deposited at the State Key Laboratory of Crop Genetics and Germplasm Enhancement, Nanjing Agricultural University.

## Abbreviations

APX: Ascorbate peroxidase
AsA: Ascorbate
BSA: Bovine serum albumin
CAT: Catalase
DHA: Dehydroascorbate
EDTA: Ethylenediaminetetraacetic acid
Fw: Fresh weight
GUS: β-glucuronidase stain
IPTG: Isopropyl β-D-1-thiogalactopyranoside
MDA: Monodehydroascorbate
PBS: Phosphate buffer solution
Pn: Net photosynthetic rate
POD: Peroxidase
rAgAPX1: recombinant AgAPX1 protein
ROS: Reactive oxygen species
rpm: revolution per minute
RWC: Relative water content
SOD: Superoxide dismutase
