# Supplementary material for: Isolation, purification and characterization of an ascorbate peroxidase from celery and overexpression of the AgAPX1 gene enhanced ascorbate content and drought tolerance in Arabidopsis
Source: BMC Plant Biol. 2019 Nov 11;19:488. doi: 10.1186/s12870-019-2095-1 (PMC6849298; doi:10.1186/s12870-019-2095-1)
Supplement: Supplementary file 1 — Additional file 1 Nucleotide acid and deduced amino acid sequence of AgAPX1 from celery. [file 12870_2019_2095_MOESM1_ESM.doc]

**Additional file 1:**

**Fig. S1** Nucleotide acid and deduced amino acid sequence of *AgAPX1* from celery.

1 atgggaaagtgctatccaattgtgagcgaggactacaaggttgctgttgaaaaatgcagaaggaagcttagaggattcatcgctgagaag

M G K C Y P I V S E D Y K V A V E K C R R K L R G F I A E K

91 aattgcgctccacttatgcttcgtctcgcgtggcactcagctggtacttatgatgttaataccaagactgggggtcctttcggaacaatg

N C A P L M L R L A W H S A G T Y D V N T K T G G P F G T M

181 aggcacaagcttgagcaatctcatgctgccaacaatggcctcgatatcgctgttaggcttttggagccttttaaggagcaattccccatc

R H K L E Q S H A A N N G L D I A V R L L E P F K E Q F P I

271 atctcttatggtgatttgtatcagttggctggagttgtttctgttgaaattactggaggtcctgatgttccattccaccctgggaggccg

I S Y G D L Y Q L A G V V S V E I T G G P D V P F H P G R P

361 gacaaagaggagccaccactggaaggccgcttgcctgatgctactttgggaaatgatcatttgagaaatgtgtttgtcaaaactatggga

D K E E P P L E G R L P D A T L G N D H L R N V F V K T M G

451 ctctctgacaaggatattgttacactttccggtggccatactctgggacgcgctcacaaggagcgttctgggtttgaaggaccctggaca

L S D K D I V T L S G G H T L G R A H K E R S G F E G P W T

541 accaaccctctcatctttgataactcttatttcacggagctcttgactggcgagaaggaaggccttcttcaattgcctactgacaaatct

T N P L I F D N S Y F T E L L T G E K E G L L Q L P T D K S

631 cttctcgaagaccctgtcttccgtccccttgttgacaaatatgctgctgatgaggatgccttctttgccgactatgcagaatctcacatg

L L E D P V F R P L V D K Y A A D E D A F F A D Y A E S H M

721 aagctatctgaacttgggtttgctgaggcctaa

K L S E L G F A E A *
