# Supplementary material for: Isolation, purification and characterization of an ascorbate peroxidase from celery and overexpression of the AgAPX1 gene enhanced ascorbate content and drought tolerance in Arabidopsis
Source: BMC Plant Biol. 2019 Nov 11;19:488. doi: 10.1186/s12870-019-2095-1 (PMC6849298; doi:10.1186/s12870-019-2095-1)

**Additional file 2:**

**Fig. S2** SDS-PAGE analysis of the purified AgAPX1 from expression in *E. coli*.

Lane1, standard markers; Lane2, total soluble protein from BL21(DE3) cells containing the AgAPX1 plasmid without induction; Lane3, total soluble protein from BL21(DE3) cells containing the AgAPX1 plasmid with IPTG; Lane 4, purified AgAPX1. The arrow indicates the AgAPX1.


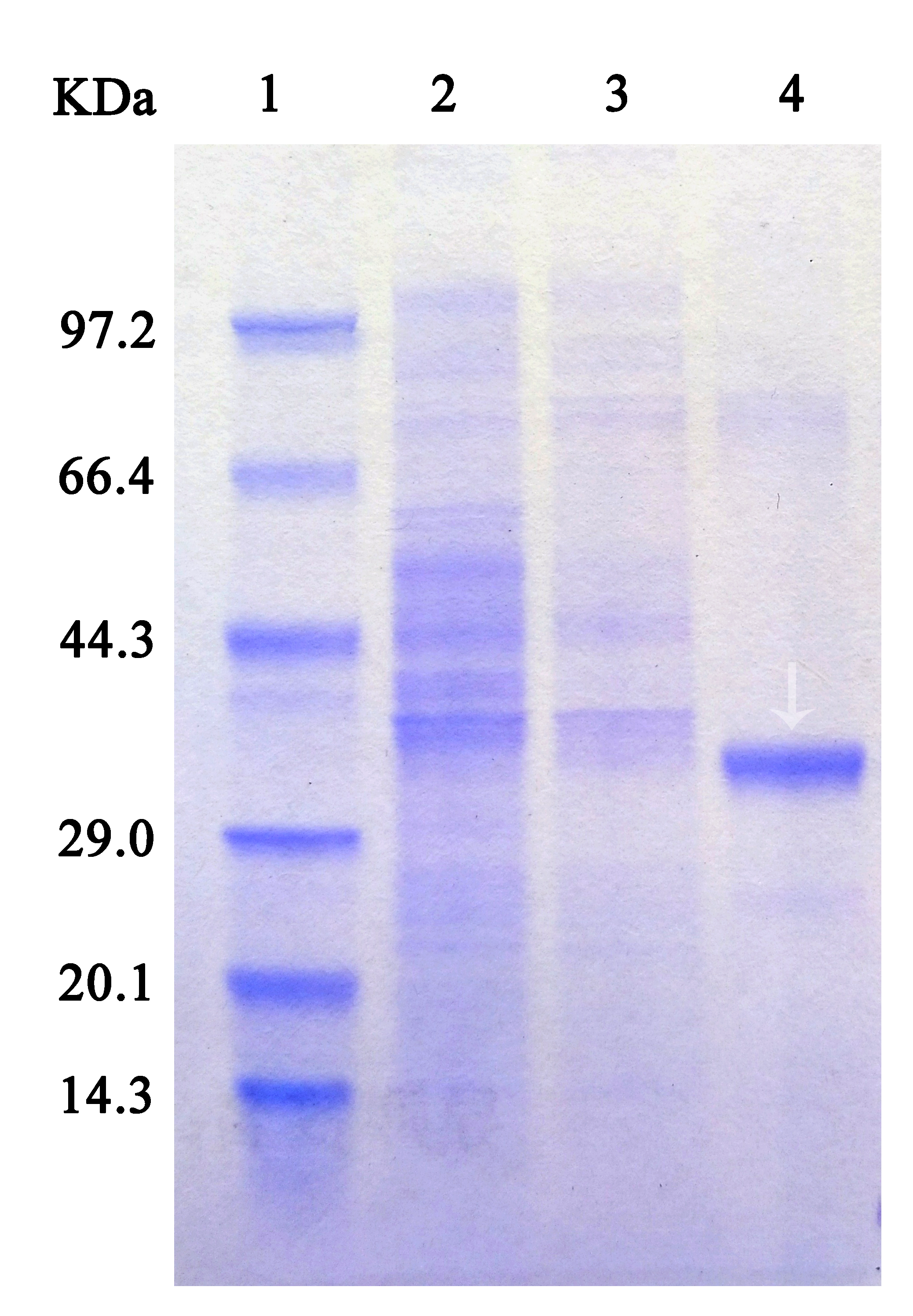

Supplement: Supplementary file 2 — Additional file 2 SDS-PAGE analysis of the purified AgAPX1 from expression in E. coli. Lane1, standard markers; Lane2, total soluble protein from BL21(DE3) cells containing the AgAPX1 plasmid without induction; Lane3, total soluble protein from BL21(DE3) cells containing the AgAPX1 plasmid with IPTG; Lane 4, purified AgAPX1. The arrow indicates the AgAPX1. [file 12870_2019_2095_MOESM2_ESM.doc]
