# Supplementary material for: Isolation, purification and characterization of an ascorbate peroxidase from celery and overexpression of the AgAPX1 gene enhanced ascorbate content and drought tolerance in Arabidopsis
Source: BMC Plant Biol. 2019 Nov 11;19:488. doi: 10.1186/s12870-019-2095-1 (PMC6849298; doi:10.1186/s12870-019-2095-1)

**Additional file 3:**

**Fig. S3** Ascorbate content in transgenic *Arabidopsis* and wild-type (WT) leaves detected by HPLC. **a** WT plants; **b** AgAPX1-4 transgenic line; **c** AgAPX1-16 transgenic line.


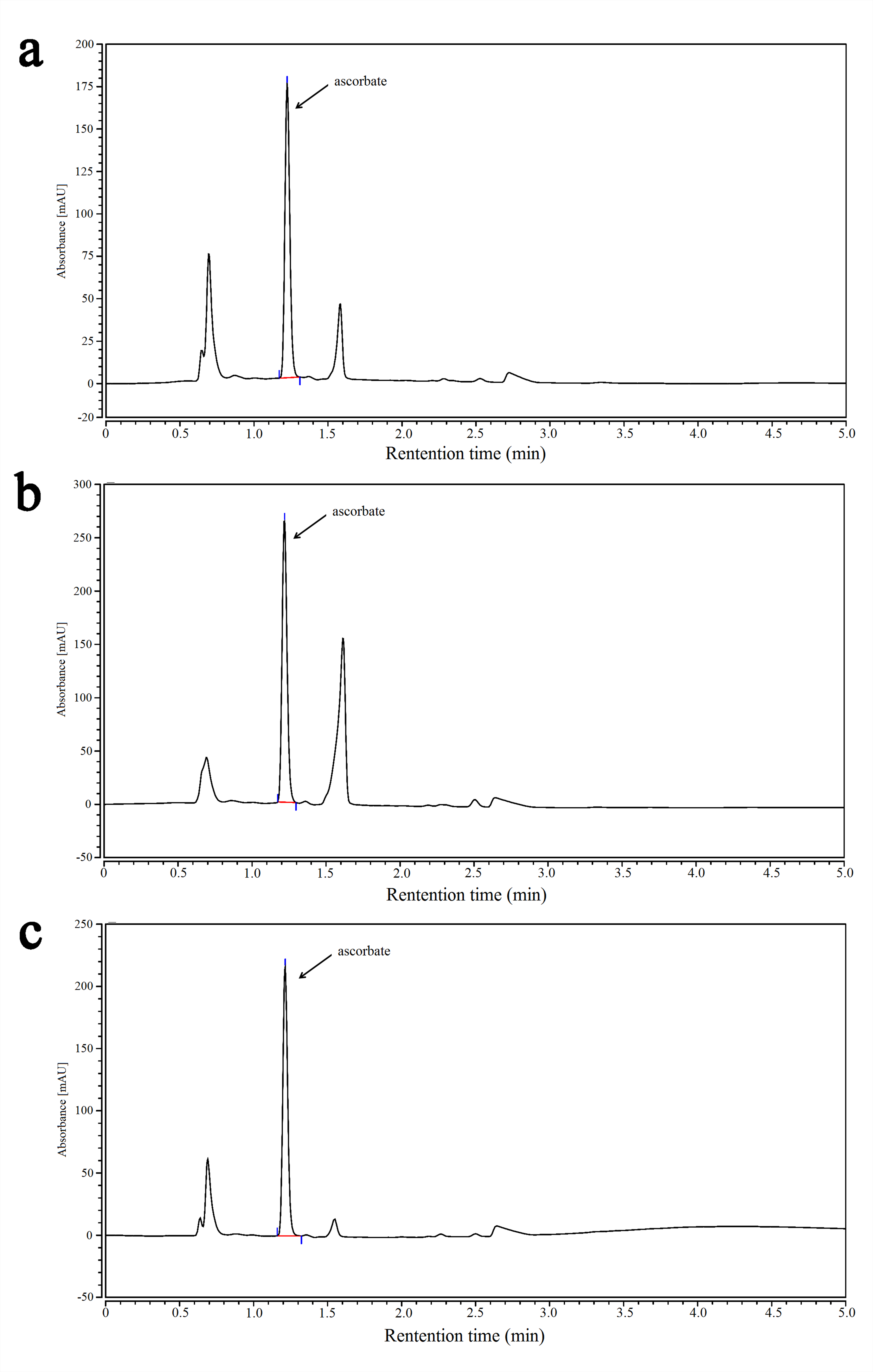

Supplement: Supplementary file 3 — Additional file 3 Ascorbate content in transgenic Arabidopsis and wild-type (WT) leaves detected by HPLC. a WT plants; b AgAPX1–4 transgenic line; c AgAPX1–16 transgenic line. [file 12870_2019_2095_MOESM3_ESM.doc]
